# Mapping Structural Connectivity Using Diffusion MRI: Challenges and Opportunities

**DOI:** 10.1002/jmri.27188

**Published:** 2020-06-17

**Authors:** Chun-Hung Yeh, Derek K. Jones, Xiaoyun Liang, Maxime Descoteaux, Alan Connelly

**Affiliations:** 1 Institute for Radiological Research, Chang Gung University and Chang Gung Memorial Hospital, Taoyuan, Taiwan; 2 Department of Child and Adolescent Psychiatry, Chang Gung Memorial Hospital, Linkou Medical Center, Taoyuan, Taiwan; 3 Florey Institute of Neuroscience and Mental Health, Melbourne, Victoria, Australia; 4 Florey Department of Neuroscience and Mental Health, University of Melbourne, Melbourne, Victoria, Australia; 5 Cardiff University Brain Research Imaging Centre (CUBRIC), School of Psychology, Cardiff University, Cardiff, UK; 6 Mary Mackillop Institute for Health Research, Australian Catholic University, Melbourne, Victoria, Australia; 7 Sherbrooke Connectivity Imaging Laboratory (SCIL), Université de Sherbrooke, Sherbrooke, Quebec, Canada

## Abstract

Diffusion MRI-based tractography is the most commonly-used technique when inferring the structural brain connectome, i.e., the comprehensive map of the connections in the brain. The utility of graph theory—a powerful mathematical approach for modeling complex network systems—for analyzing tractography-based connectomes brings important opportunities to interrogate connectome data, providing novel insights into the connectivity patterns and topological characteristics of brain structural networks. When applying this framework, however, there are challenges, particularly regarding methodological and biological plausibility. This article describes the challenges surrounding quantitative tractography and potential solutions. In addition, challenges related to the calculation of global network metrics based on graph theory are discussed.

UNDERSTANDING THE BRAIN in terms of structural and functional networks has become one of the frontier topics in neuroscience; this is evidenced by considerable investment internationally to prompt the innovation of advanced neuroimaging techniques, to enable identification of the human *connectome*, i.e., the comprehensive description of brain structural or functional connections.^1–4^ There has been a rapid rise in activity in the field of *connectomics*, where researchers seek to discover the neural substrates underlying cognition and behavior, in either healthy or diseased states.^1,5–12^

As the human brain has a dense neural architecture comprising billions of neurons to form one of the most complex network systems in the world, it is an outstanding challenge to obtain its connectivity patterns in vivo with the elements and connections in different levels; for instance, at the microscale (single neurons), mesoscale (a group of neurons), and macroscale (distinct brain gray matter (GM) regions).^3,4,13^ Modern noninvasive neuroimaging modalities have enabled both functional and anatomical connectivity information to be measured in the living human brain (Fig. 1). Among those techniques, diffusion magnetic resonance imaging (MRI) is the main in vivo technique for inferring white matter (WM) fiber connectivity due to its noninvasive ability to delineate WM pathways in the brain, using so-called fiber-tracking or tractography.^14^ To date, diffusion MRI-based tractography has become an essential component of the field of connectomics, for the investigation of WM connectivity in the healthy brain,^15–17^ as well as how connectivity is disrupted by brain disorders.^18^

Much effort toward investigating human brain connectomics focuses on the application of graph theoretical analysis, which provides a range of metrics that characterize the topology of the network.^19^ Such metrics facilitate explorations of the information integration, segregation, and propagation in the brain. With these approaches, researchers have found a nonrandom architecture of brain networks, such as small-worldness,^20^ efficiency,^21^ modularity,^22^ network hubs,^23^ and rich-club organization.^24^

This review focuses on specific technical challenges and issues in the construction of tractography-based connectomes using diffusion-weighted images (DWIs), and on the validity of the commonly-used graph theoretical analysis workflow to analyze network properties from the derived structural connectomes. The remaining sections of this article are organized as follows:

- **Building an Individual’s Connectome From DWIs**—presents a general overview of a processing pipeline used for processing diffusion MRI data (page 3).
- **Tractogram Generation**—discusses two types of known tractography biases, namely, subsection *Streamline Termination Bias* (page 4) and subsection *Streamline Quantification Bias* (page 5). Advanced tractography techniques that aim at tackling these sources of bias will be introduced, and then the outcomes of subsequent graph theory analysis following the application of these existing methods will be discussed (subsection *Effects of Bias Correction on Downstream Connectivity Analysis*; page 6).
- **Connectome Construction**—focuses on decisions that need to be made in the course of connectome construction. These include the choice of a brain parcellation scheme to define brain regions-of-interest (ROIs) (*Defining Nodes*; page 6); the definition of inter-areal connectivity (*Defining Edges*; page 8); the mechanism to associate streamlines with brain GM ROIs (*Streamline-to-Node Assignment*; page 9), and then followed by the influence of the *Disparities Between Tissue Segmentation and Brain Parcellation* (page 9) on the efficacy of connectome generation; finally, the need for *Assessing Reproducibility of Connectome Construction* (page 11).
- **Connectivity Analysis Using Graph Theory**—begins with a discussion of the validity and potential implications of performing some processing steps on tractography-based connectomes, such as applying a threshold and/or binarizing the connectome, covered in the subsection *Structural Connectome: Binary vs. Weighted (page 12) and Weighted Structural Connectome: Dense vs. Sparse* (page 12). Then the section provides the authors’ viewpoints on the computation of weighted graph theory metrics and other topological properties, including a discussion of the role of quantitative tractogram processing in connectomics research (subsection *Graph Theoretical Analysis: From Binary to Weighted Metrics*; page 13). The section ends with some remarks on the analysis and interpretation of group differences in connectomics metrics (subsection *Every Bias Correction Matters*; page 14).
- **Summary**—highlights challenging issues and recommended strategies in structural connectome and highlights future perspectives and demand in this rapidly growing field (page 14).

## Building an Individual’s Connectome From DWIs

A connectome is a full connectivity diagram of the brain network and can be derived from functional or structural data.^1,3^ Mathematics and computing domains often use the term “graph” to depict the connections and interactions of a network. A graph is composed of a set of nodes (or vertices) that are connected by edges (or connections). The edges can be directed (edges are directed from one node to another) or undirected (no direction for each edge), and additionally can be unweighted/binary (i.e., an edge exists or not) or weighted (where edges have varying “strengths”). A simple way to represent a connectome/network/ graph is by using a 2D matrix representation, the so-called adjacency matrix or connectivity matrix.

At macroscopic scales, a structural connectome aims at mapping long-range WM fiber connections between distinct cortical and subcortical GM areas in the brain. In the context of diffusion MRI, building an individual’s structural connectome is done by using the result of whole-brain streamline tractography (i.e., the tractogram) to link the ROIs defined by a brain parcellation scheme, thereby inferring potential WM connections between pairs of GM areas.^25^ The GM ROIs and the *inferred* WM connections^[1]^ are used as nodes and edges respectively to construct the connectome.^2,26^ Currently, tractography-based structural connectomes are undirected, as diffusion MRI alone cannot differentiate whether a pathway is afferent or efferent. This is because diffusion is a symmetrical process, i.e., the probability of molecular displacement along a vector is the same as that along the antipode.^27^

**Figure 2:**
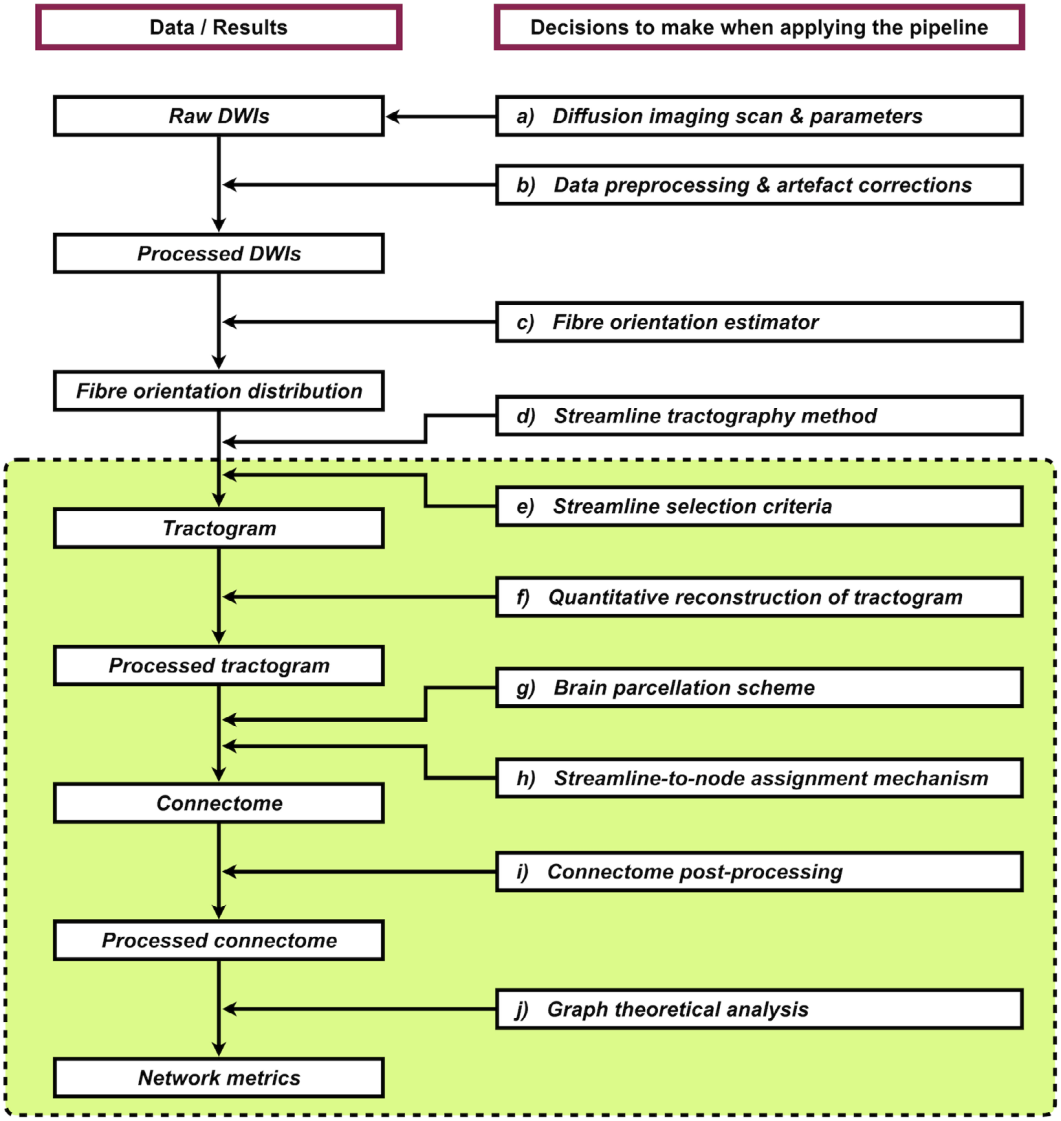
An example processing workflow for generating an individual’s structural connectome using diffusion MRI data (an expanded version of the left branch in Fig. 1). Left column: Each box denotes the raw, interim, or final products of this pipeline. Right column: Each box describes the class of data processing involved in this pipeline. Within each procedure, there are many relevant options and parameters that have to be considered, where each choice can potentially affect the final output network metrics and the inference drawn from this technique. This shows the complexity of data processing in tractography-based structural connectomics research. The green box indicates the processing steps that are specifically discussed in the present article.

As mentioned above, building a structural connectome from an individual’s DWIs is straightforward conceptually. In practice, a wide range of practical decisions have to be made prudently when taking this approach. These include (but are not limited to): a) imaging sequence and parameters; b) DWI preprocessing and artifact corrections; c) fiber orientation estimator; d) streamline tractography method; e) streamline selection criteria; f) quantitative reconstruction of the tractogram; g) brain parcellation scheme; h) streamline-to-node assignment mechanism; and i) connectome post-processing (see also Fig. 2 for the overall workflow). Some crucial methodological details might not be appropriately handled by the end-users; others are sometimes hidden in the automated processing script (or the “black box”), of which end-users may not be fully aware. Researchers focusing in other domains (such as clinical, theoretical, or computational neuroscience) may work directly on the final product of these procedures, i.e., the connectivity matrices. However, although many techniques have been developed to ameliorate many of the challenges with tractography/connectome reconstructions, they are not always widely adopted by the community in practice, meaning the results are suboptimal.

Recent articles have covered various aspects of the limitations and factors that can affect tractography and connectome results.^17,27–33^ The scope of **Tractogram Generation** and **Connectome Construction** section is to discuss some specific challenges (points (e-h) above), where those factors have significant influences on the outcomes of structural connectivity analysis, particularly for the application of graph theoretical approaches.

## Tractogram Generation

This section describes two known sources of tractography biases that can have significant flowthrough influences on connectomics results, as well as methods that have been proposed to ameliorate them.

### Streamline Termination Bias

Typical diffusion MRI data do not contain any information regarding the possible location of cell bodies, but only provide an ensemble signal averaged over a voxel that has a much larger scale than a single neuronal cell. Hence, there is no explicit biological indicator of where a reconstructed streamline should terminate. Conventional tractography algorithms often terminate streamlines when they travel into voxels of low diffusion anisotropy (e.g., fractional anisotropy) or low amplitude of the fiber orientation distribution, or when they need to make a sharp turn to continue (i.e., high curvature).^17,34^ However, these criteria are often insufficient to warrant appropriate streamline termination and often cause streamlines to end within WM or cerebrospinal fluid (CSF), or even to propagate through and beyond the cortical GM; all such results are biologically implausible.^35^ As such, it is necessary to introduce additional biologically-relevant constraints (where reasonable) to ensure that the reconstructed streamlines are plausible delineations of WM connections.

The most common way to constrain the tractography results is using so-called targeted tracking (or track editing/ virtual dissection) approaches.^36–38^ Typically, this involves defining reasonable inclusion or exclusion regions (or “waypoints”) based on prior anatomical knowledge to serve as additional streamline selection criteria, i.e., the inclusion and exclusion regions are used to determine whether a streamline should be selected or not. Targeted tracking is, however, feasible only for well-defined WM pathways in the brain, for which that prior anatomical knowledge exists, and in which each pathway usually requires multiple inclusion and exclusion regions for effective reconstruction. It is therefore difficult to apply this technique in the context of connectomics, where whole-brain fiber-tracking is preferable for investigations of any potential WM connectivity.

**Figure 3:**
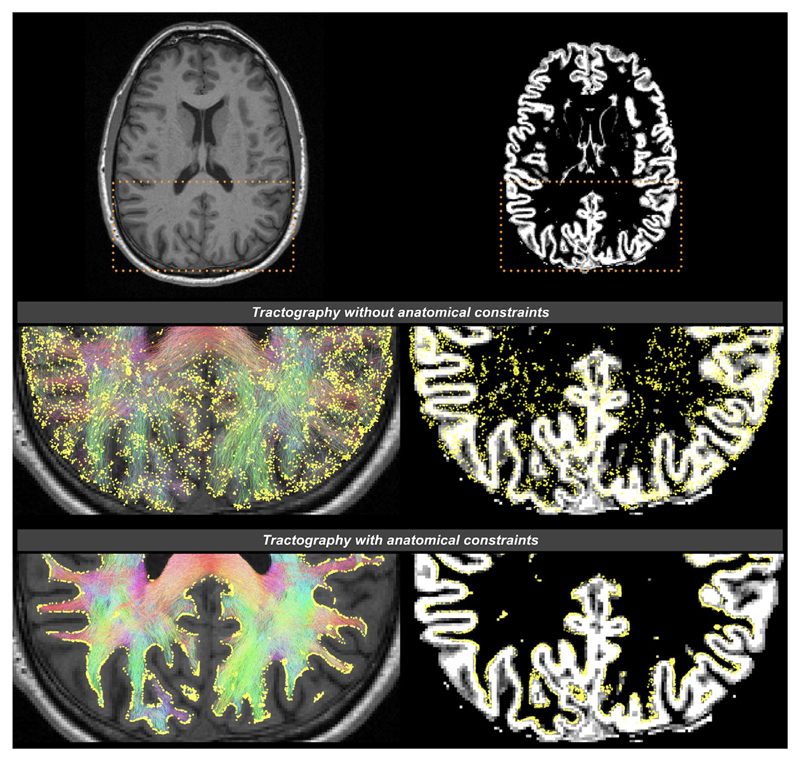
The effects of applying anatomical constraints on diffusion MRI streamlines tractography, shown on a transverse slice image of a human brain. The background images are a structural T1-weighted image on the left column, and the corresponding gray matter partial volume map following tissue segmentation on the right column. Streamlines are color-coded according to their orientations (red: left–right; green: anterior–posterior; blue: inferior–superior). The yellow spheres are the streamline endpoints. Top row: The dashed boxes indicate the zoomed brain areas shown in the middle and bottom rows. Middle row: Streamlines generated without anatomical priors; streamline endpoints distribute throughout the brain. Bottom row: Streamlines generated with anatomical priors; streamline endpoints only occur at the interface between GM and WM (demonstrated using Refs. 35, 42). It has been revealed that the considerable improvements in such streamline terminations provided by the use of anatomical constraints have significant influences on structural connectivity patterns and the outcomes of connectomic metrics.^43,44^

As mentioned previously, diffusion MRI data do not provide information about cell bodies or synapses to guide tractography terminations; nevertheless, there are still some fundamental assumptions we could make regarding the required characteristics of any estimated streamline connections generated from the data. For example, we could consider basic principles of how the neurons are arranged in the brain: the axons emanate from the cell bodies within the GM and ultimately connect to other cells, either elsewhere within the brain or elsewhere in the body. This knowledge allows us to impose the following anatomically-relevant constraints to ensure they are consistent with the nature of WM fibers, including: a) fibers should reach at least the interface of GM and WM at both ends; b) fibers do not terminate either in the middle of WM or in CSF. This is the rationale behind the so-called anatomically-constrained tractography^35^ or alternatives^39–41^; anatomical priors can be obtained from tissue segmentation or surface reconstruction of high-resolution anatomical MR images (usually T_1_-weighted images), and can be incorporated into the fiber-tracking process for streamline selection (Fig. 2e). This class of methods prevents biologically unrealistic connections by discarding streamlines that do not match those a priori assumptions above, as well as constrain streamlines terminations to occur only at the interface between GM and WM, within the subcortical GM, or at the spinal column. These techniques are increasingly being adopted, particularly in whole-brain tractography, as they can greatly improve the robustness and biological plausibility of streamline generation (see Fig. 3 for an illustration of the effects of these methods on tractography outcomes).

Note that the references to pathways being potentially “biologically meaningful” are based on the aforementioned constraints/assumptions indicating which streamlines *cannot* represent biology, but not on any assertion that any given streamline is a “true positive.” For example, it is reasonable to suggest that since axons do not terminate in the middle of WM, then a streamline that does this cannot be representative of any biological connection. It does not imply the opposite, i.e., that any streamline that does connect two GM regions must be real—only that if it does not do this then it cannot represent a connection within the brain.

### Streamline Quantification Bias

A number of challenges associated with quantification of the structural connectome based on a whole-brain tractogram have been identified (see Refs. 27, 30 for review). One of the underlying issues is that within an MRI voxel, the reconstructed streamline count (or density) does not represent the actual fiber density, which means that “raw” streamline-based connection densities are not biologically relevant.^45^ Thus, using the number of streamlines from such a “raw” tractogram reconstruction cannot serve as a valid biomarker of connectivity strength between the connected areas.^27,30^

One well-recognized quantification bias of the reconstructed streamline density comes from the “length” effect of WM fiber pathways: 1) streamlines could be over-reconstructed in longer pathways when homogeneously initiating streamlines (i.e., seeding) throughout WM voxels^45,46^; notably, such a seeding strategy is still commonly used in neuroscience applications; 2) streamlines could also be underestimated in longer pathways, which are more difficult to track due to the increased number of tracking steps required (i.e., the number of sampling points).^27^ Both scenarios will inevitably compromise further connectomic analysis of brain networks when a connectome is weighted or filtered based on streamline density.

**Figure 4:**
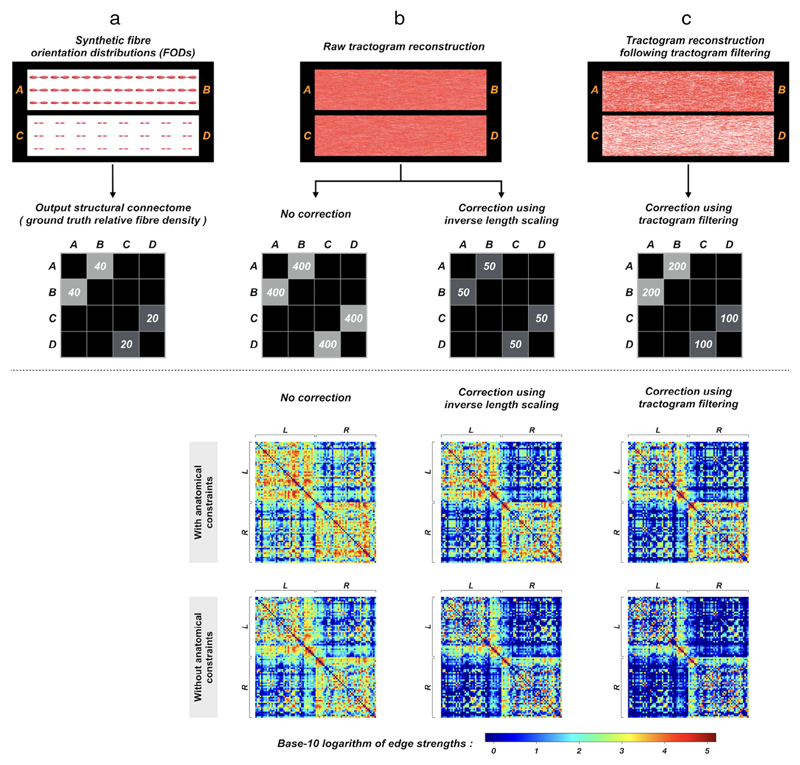
The effects of quantitative tractogram postprocessing on subsequent quantification of connectome construction, illustrated using a synthetic example (upper panel) and human image data (bottom panel): Upper panel: (a) Fiber bundles A$B and C$D are simulated using synthetic fiber orientation distributions (FODs), where the size of FODs (i.e., apparent fiber density^58^) in A$B is twice that in C$D. The relative fiber density of this synthetic FOD field is reflected in the connectome. (b) When running fiber-tracking, no matter whether seeding is performed uniformly from the whole (i.e., mimicking WM seeding) or from the extremities (i.e., mimicking seeding at the interface between GM and WM) of the fiber bundles, the same number of streamlines will be generated in both pathways. Obviously, the results do not comply with the synthetic ground-truth. The resultant connectome edges weighted by streamline density are therefore also biased in A$B and C$D when no correction is applied. Also, as inverse length scaling does not consider the size of FODs, it cannot correct for this type of quantification bias. (c) With the application of quantitative tractogram processing techniques (e.g., using Refs. 42, 45), the reconstructed streamline densities are rendered consistent with the underlying FOD field in A$B and C$D, making the connectome edges weighted by streamline density linearly proportional to the actual fiber density. Bottom panel: Group-averaged connectivity matrices generated from a cohort of healthy subjects. Probabilistic tractograms are generated with WM seeding; nodes are defined by the Desikan–Killiany atlas^48^; edges are defined by streamline count. The first row shows the connectomes obtained from tractograms with anatomical constraints and then processed by different levels of quantitative bias correction (left: no correction; middle: correction using inverse length scaling; right: correction using tractogram filtering). For comparisons, the bottom row shows the results without anatomically-constrained tracking. L and R denote left and right hemispheres, respectively. The differences among these connectivity matrices are clearly visible, and indeed a range of popular connectomic metrics are significantly different.^43^ This figure is adapted from Ref. 43 with copyright permission.

One way to bypass the first scenario is by seeding uniformly from the interface between GM and WM instead of from WM regions, which however does not resolve the latter issue. Alternatively, a popular heuristic approach to compensate for the former is by scaling the contribution of each streamline to the relevant connectome edge by its inverse length,^2^ i.e., a longer streamline receives a smaller weight. But since the second scenario is contradictory to the first one, it is difficult to address both cases simultaneously via scaling by length heuristic.^46^ Although more dedicated length correction methods (e.g., Ref. 47) could be applied, they are still limited to addressing one specific type of bias that arises from the length of fiber pathways and are far from satisfactory in many other cases (see Ref. 27 and the synthetic example in Fig. 4), providing therefore an incomplete correction of whole-brain tractogram/connectome quantification.

More recently, advanced tractogram post-processing methods have been proposed to match the consistency between the signal *contributed or predicted* by the streamlines passing through the voxel, and the actual MRI signal *observed* at that voxel^45,49–57^ (Fig. 2f). This class of “tractogram filtering” or “microstructure-informed tractogram processing” technique modulates the contribution of streamlines to the MRI signal driven by local tissue microstructure properties (e.g., apparent fiber density (AFD)^58^), thereby making full tractograms more biologically relevant^59^ and are thus particularly important for the quantification of structural connectivity. While differing in their algorithms, these techniques are designed to address a wider range of tractography biases, as compared to, for example, the heuristic approach based on inverse length scaling that does not compensate for the same range of potential biases in streamlines reconstruction, thus resulting in a correction of the tractogram that is incomplete (as shown in Fig. 4).

### Effects of Bias Correction on Downstream Connectivity Analysis

It has been demonstrated that the characteristics of structural connectomes, including many widely-used global network metrics obtained from graph theoretical analysis are significantly sensitive to fiber-tracking biases as well as degrees of bias correction.^43^ This suggests that the level of tractography bias correction may alter the fundamental interpretations of connectivity. While there is no ground-truth data to indicate which correction method yields the most accurate network characteristics, nevertheless it is still reasonable to conclude that the more reliable metrics are those resulting from more comprehensive tracking-bias correction strategies. In addition to biological accuracy, assessing the reliability/reproducibility of the processing pipelines is also crucial ahead of their applications^60,61^ (discussed in subsection *Assessing Reproducibility of Connectome Construction*; page 11).

## Connectome Construction

### Defining Nodes

**Figure 5:**
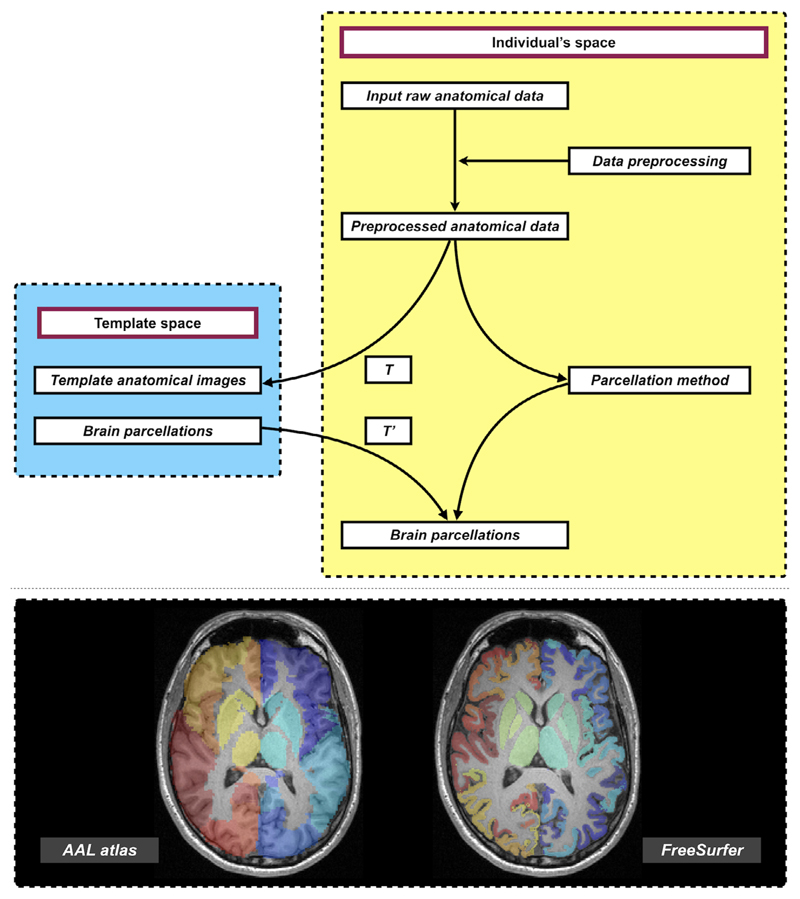
Upper panel: An overview workflow of common approaches to prepare brain parcellation images in individual space. Following pre-processing of structural image data (usually T1-weighted images), individual’s brain parcellations can be obtained via: (a) applying brain cortical reconstruction and parcellation techniques (right branch), or (b) warping the brain atlas typically defined in standard space into individual space (left branch). The general procedure of the latter includes computing image transformations (denoted as T) by registering an individual’s anatomical images to those provided in the template space, and then pull the atlas from the template space to individual space via inverse transformation (denoted as T’). Bottom panel: Examples of brain parcellations transformed from a template/standard space into an individual’s space (left, e.g., automated anatomical labeling (AAL) atlas^62^), or generated directly in the individual’s space via brain parcellation techniques (right, e.g., FreeSurfer parcellation^68^; Desikan–Killiany atlas^48^). Brain parcellations are overlaid on the structural T1-weighted images.

For studying structural brain networks, network nodes are typically obtained from brain parcellation of anatomical MRI data. Various types of parcellation techniques have been proposed to identify homogeneous brain GM areas to form the ROIs,^48,62–67^ i.e., the rows and columns of the connectivity matrix. These ROIs are typically derived either through a sophisticated processing pipeline directly applied to an individuals’ image data, or through transforming a pre-defined atlas to label an individual’s image data into distinct areas (see also Fig. 5). Brain parcellation is in itself methodologically challenging and an active research field. Studies have shown that the topological characteristics of structural connectomes can be significantly influenced by the choice of parcellation scheme,^69^ yet there is no consensus regarding which brain parcellation schemes could be optimal for constituting the nodes of the brain networks.

### Defining Edges

Currently, there is no consensus on what serves as a good measure of “connectivity” between nodes. There are many options, and studies usually have their own choices and interpretations. On the one hand, the connectivity or edge can be defined by the number, length, volume, or probability of all streamlines between the corresponding nodes. On the other hand, it can also be defined by the mean values of a diffusion metric within the volume along the path of streamlines between the interconnecting nodes; the diffusion metric can be obtained from the diffusion tensor model (e. g., apparent diffusion coefficient, fractional anisotropy, axial and radial diffusivities),^70^ from other models such as neurite orientation and dispersion density imaging (NODDI; e.g., using intracellular volume fraction)^71^ or AFD^58^ from constrained spherical deconvolution (CSD),^72,73^ or other techniques such as g-ratio mapping,^74^ or methods combining multiple contrasts.^75,76^ After connectivity has been defined, there is also considerable diversity on how “connectivity” has been post-processed. Some studies have “binarized” the edges and worked on the “unweighted” connectivity (discussed in subsection *Structural Connectome: Binary vs. Weighted*; page 12). Many studies have pruned the connectomes by applying thresholds to filter out weak connections (discussed in subsection *Weighted Structural Connectome: Dense vs. Sparse*; page 12).

The connectome would in principle be more biologically meaningful if the metric used to define the “connectivity” has physical or biological meaning.^77^ This would also allow subsequent weighted graph theoretical analysis to be more compatible (see subsection *Graph Theoretical Analysis: From Binary to Weighted Metrics* for more details; page 13). As described in the Tractogram Generation section above, modern tractography techniques are designed to achieve this aim: anatomically-constrained tracking respects the anatomy for selecting appropriate streamlines; quantitative tractogram filtering techniques are particularly relevant in this aspect, as streamline reconstruction is associated with biologically-relevant and quantitative features such as AFD.^58^ These methods allow the number of streamlines to be a potentially reasonable metric to represent connectivity.

### Streamline-to-Node Assignment

The construction of a structural connectome typically requires a streamline-to-node assignment mechanism to associate streamlines with GM ROIs or nodes (Fig. 2h). It has been recently demonstrated that the strategy by which individual streamlines are terminated and subsequently assigned to particular edges in a connectome can have a significant impact on the connectome characteristics, including some of the most commonly-used graph theoretical metrics as well as the modular connectivity patterns.^44^ The convoluted interactions among the spatial extent of labeled brain ROIs, streamline termination and selection criteria, and streamline-to-node assignment mechanism can have nontrivial substantive consequences for connectome quantification. Importantly, their impact has been reported in both clinical-grade MRI data and the high-quality data from the Human Connectome Project.^78,79^ Therefore, future tractography-based connectomics studies should not overlook the relevant influences involved in the process of streamline-to-node assignment, and the exact strategy used to generate a connectome should be stated explicitly.

**Figure 6:**
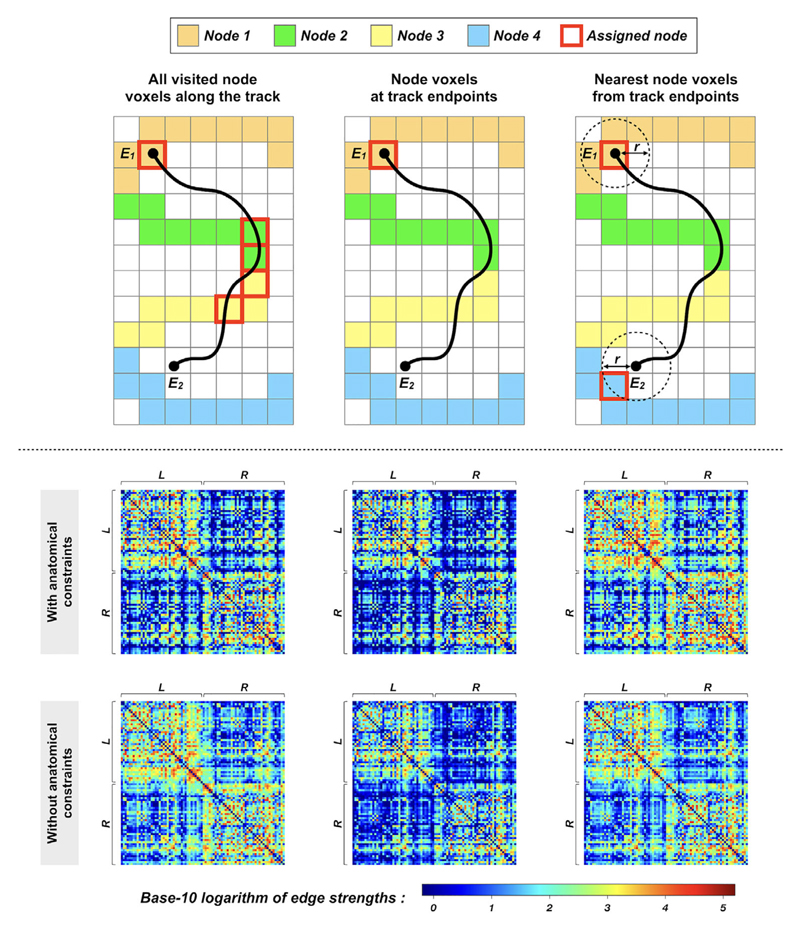
The effects of streamline-to-node assignment mechanisms on connectome construction, illustrated by a toy example (upper panel) and human image data (bottom panel): Upper panel: Examples of mechanisms used to assign streamlines to network nodes—streamlines shown in black; streamline endpoints denoted as E1 and E2; network nodes shown as colored voxels. The colored voxels added with red borders indicate the assigned nodes. As shown in this figure, even for an identical streamline, the assigned nodes (and therefore the outcome connectivity) among those streamline-to-node assignment methods are all different, suggesting that the design of such a mechanism can have direct influence on connectome quantification (see Ref. 44 for detailed explanations). Bottom panel: Group-averaged connectivity matrices generated from a cohort of healthy subjects. Probabilistic tractograms are generated; nodes are defined by Desikan–Killiany atlas^48^; edges are defined by weighted streamline counts as provided by quantitative tractogram processing.^55^ In the upper row, the matrices are obtained using tractography with anatomical constraints, where the three streamline-to-node assignment mechanisms shown in the upper panel are used for connectome construction. For comparisons, the bottom row shows the results without anatomically-constrained tracking. L and R denote left and right hemispheres, respectively. The outcome connectivity pattern and connectomic metrics are significantly different among these connectivity matrices,^44^ highlighting the necessity of selecting an appropriate strategy for connectome construction. This figure is adapted from Ref. 44 with copyright permission.

The lack of such an explicit description in the majority of connectomics articles might be due to the fact that there has been no clear consensus on how to address this fundamental problem. Many studies applied a mechanism in which all labeled node voxels intersected along the trajectory of a streamline are considered connected (see Fig. 6 for illustration). This approach allows a streamline to contribute to multiple edges in the same connectome. Not only does this facilitate many inherently unreliable indicators of connectivity, but the limitations of current tractography techniques are simply not able to track fibers within and between multiple GM regions in a robust manner, making the resultant connectome quantification untrustworthy and lacking in biological plausibility.

Under the challenges of present tractography methodology, it is more sensible to assign a given streamline to exactly two GM regions for structural connectome construction. This is compatible with what we are fundamentally trying to achieve when calculating metrics of connectivity: given a basic understanding of how neurons are arranged in the brain—an axonal fiber connects two cell bodies in the GM—the ideal case therefore is that streamlines connect one GM node with another to represent a potential neuron–neuron connection. It is worth noting that this consideration is closely aligned with the general constraints adopted in anatomically-constrained tracking techniques^35,39–41^ that are designed to ensure biologically meaningful pathways (as described in subsection *Streamline Termination Bias*; page 4-5). The appropriate streamline termination given by these methods facilitates the streamline-to-node assignment process and is therefore beneficial for identifying connectivity between GM ROIs.

### Disparities Between Tissue Segmentation and Brain Parcellation

**Figure 7:**
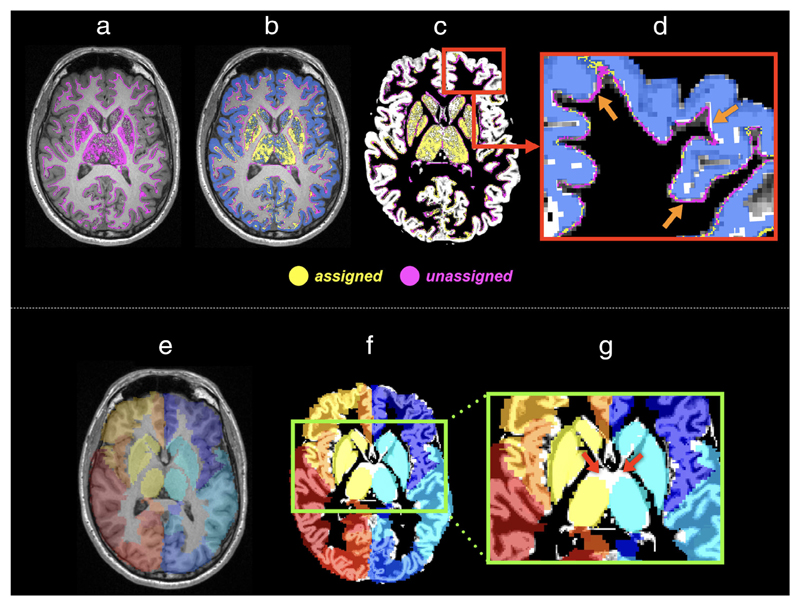
The effects of the misalignment between image-intensity-based tissue segmentation and brain parcellation on the assignment of streamlines to network nodes: Upper panel: (a) With the application of anatomical constraints (e.g., Ref. 35), streamline endpoints (colored in purple) occur at the GM–WM interface or within the subcortical GM. (b) Due to factors such as discretization of structural labels, many of these endpoints (purple points) do not locate inside the, for example, FreeSurfer parcellation image (blue ribbon)^48^ and thus are not assigned to a label. (c) The T1 image shown in (a,b) is replaced by the GM partial volume maps derived from intensity-based tissue segmentation.^80^ (d) A zoom region of (c) illustrates the discrepancy (pointed by arrows) between tissue segmentation and brain parcellation, revealing that streamlines cannot be assigned purely based on the voxels where the endpoints reside. Bottom panel: The discrepancies also present in subcortical GM, and in fact could be even crucial when parcellation images are prepared by transforming an atlas to an individual’s space (i.e., the left branch of Fig. 5). This is because the degree of misalignment between subcortical GM segmentations and brain parcellations could be increased by the registration error. As an example: (e) The AAL atlas is coregistered to individuals’ data via linear and nonlinear transformations. The background shows a T1-weighted image slice of the subject. (f) The background image is replaced by the GM partial volume map obtained from tissue segmentation. (g) The red arrows point to the considerable misalignment between two images at the thalami regions.

Combining anatomical priors into the tractography framework is an effective way to remove parts of false streamline connections in whole-brain tractograms. The most common approach to doing this involves segmentation on structural T_1_-weighted images to obtain tissue partial volume fractions of WM/GM/CSF (e.g., using FSL’s FAST algorithm^80,81^) in each voxel, from which the boundary of GM and WM is estimated and streamlines are terminated at this boundary.^35,39^ This image-based boundary, however, is not always compatible with that derived from brain parcellation atlases, where images contain only discrete integers labeling distinct brain ROIs (i.e., without considering tissue volume fractions). Such inconsistencies between tissue segmentation (used for tractography) and brain parcellation (used for connectome construction) can result in substantially biased connectomes (see Fig. 7).^44^

Currently, a heuristic fix to mitigate the effects of such an acknowledged discrepancy problem is by assigning streamline endpoints to the nearest labeled voxel of the parcellation images, within a reasonable distance.^59^ Typically, this distance is confined to be within a local area (e.g., ~2 mm) around the endpoints of any given streamline in order to compensate for small discrepancies between terminating in a voxel (derived from tissue partial volume maps) and the GM boundary (derived from parcellation images) being defined as starting or ending in an adjacent voxel.

Due to the partial volume effect inherent to the image data resolution, it is a challenge to extract the exact location of the border between different tissue types. Each segmentation algorithm may operate differently, leading to variations in the output images. One way to ensure that the algorithms applied to streamline termination and brain parcellation are exactly compatible is to use a surface-based approach,^40,41^ where tissue surfaces with structural or functional labels are used at both tractography and connectome construction stages. This can minimize uncertainties induced by additional mechanisms typically required in the image-based approaches for assigning streamlines to brain parcels.

### Assessing Reproducibility of Connectome Construction

In addition to ensuring that the connectivity data derived from diffusion MRI-based tractography are biologically relevant (as discussed above), and therefore constitute appropriate input data for subsequent connectomic analysis, it is also important to consider the effects of any choices made on the reproducibility of subsequent analyses.^60,61^ Since there are various options and parameters available for the construction of a structural connectome, it is important to test the stability and reproducibility of data resulting from the selected strategy, such as the choice of diffusion MRI acquisition scheme, fiber orientation estimator, tractography parameters, and brain parcellation scheme. With the prevalence of graph theoretical metrics in connectomics research, most evaluations have been conducted on the reproducibility of graph theoretical metrics.^82–89^ It is worth mentioning that while the stabilities of these metrics could be informative for guiding the selection of a connectome construction pipeline, it would also be important to assess the reproducibility directly on the connectivity matrices themselves (e.g., Refs. 40, 59, 90), rather than at the level of derived metrics, as they are indirect representations of a connectome.

## Connectivity Analysis Using Graph Theory

Graph theory is a powerful mathematical analysis framework for modeling and quantifying the topological characteristics of network systems.^91^ To date, the majority of brain connectomics research has applied graph theoretical analysis to investigate group differences in local or global graph theoretical measures. The main advantage of graph theoretical approaches is that they quantify properties of the network by using summary metrics.^19^ Each metric captures one aspect of the complex topology and is convenient for statistical analysis and reporting results. By applying graph theoretical analysis on diffusion MRI-based structural connectomes, studies have demonstrated the nonrandom and modularized organization of structural networks in the human brain.^5,22^ Studies have also shown changes in brain network structures during normal or abnormal development, aging, and in diseased states.^18,92,93^ Furthermore, structural network properties have been found to correlate with behavioral or cognitive functions,^94,95^ suggesting their associations with functional dynamics. All these demonstrate that graph theoretical methods are a promising way to advance our understanding of architecture, coupling, and evolution of brain networks, as well as providing novel insights into biological mechanisms of clinical disorders.

Graph theoretical analysis has been applied extensively in the field of brain connectomics. However, on top of the technical challenges highlighted in the previous sections related to the reliability of data derived following structural connectome construction, there are challenges that require further consideration when applying graph theory to the analysis of the connectome data generated from tractography. Some practical issues are discussed in the following sections.

### Structural Connectome: Binary vs. Weighted

When constructing connectomes, it is crucial that the connectivity between brain regions is as representative as possible of the underlying anatomy. Given various biases involved in conventional tractography, it has been well recognized that the raw streamline count or density is not a valid metric for quantifying inter-areal connectivity “strength” of WM fibers.^27,30^ For this reason, previous studies often impose some post-processing steps on the connectomes in order to bypass the inherent quantitative inaccuracy of streamlines tractography. Some have gravitated towards using binary connectomes, where streamlines are used merely to indicate the existence or absence of inter-areal connections. Typically, the way to binarise a connectome is by selecting a threshold such that all edges smaller than the threshold are set to zero, otherwise set to one. Determining an appropriate threshold, however, is not straightforward and often arbitrary. Importantly, such a binarized version may be an oversimplification, since the connectivity patterns cannot reflect the known heterogeneous distribution of fiber connection densities demonstrated in tracing studies (e.g., Ref. 96). Since this knowingly artificial binarization process will unfavorably result in connectivity patterns that deviate from realistic biology at an early stage of the network analysis (Fig. 2i), it is challenging to justify that subsequent graph theory measures remain biologically plausible.

By contrast, a “weighted” structural connectome should in principle provide a more powerful characterization of the biological properties,^97^ if the inter-areal connection densities derived from streamlines tractogram can be made to represent the underlying features of WM fiber connectivity accurately. This is actually the aim that quantitative tractography techniques, such as tractogram filtering or microstructure-informed tractogram reconstruction, aspire to achieve (see subsection *Streamline Quantification Bias and Effects of Bias Correction on Downstream Connectivity Analysis* above; page 5-6). With the advances of these tractogram processing methods, the primary argument for using binary con-nectomes, namely, the non-quantitative nature of streamlines tractography, is no longer valid. Hence, weighted con-nectomes are highly recommended and increasingly adopted (e.g., Refs. 75, 76, 98–100) for structural connectivity analysis as they can encode more biological features and therefore offer more biologically meaningful information than binary versions. This is particularly relevant in the context of many conditions in which there are neurological deficits associated with reduced connectivity densities rather than the complete absence of affected fiber tracts (e.g., in motor neuron disease,^58^ glaucoma,^101^ epilepsy,^102^ Alzheimer’s disease^103^); binary connectome studies would be ill-suited to such circumstances.

### Weighted Structural Connectome: Dense vs. Sparse

There is currently no consensus on how to extract topological measures from weighted structural connectomes properly. In spite of this, it is a common practice in the field to filter out weak connections (via thresholding) that are more likely to be induced by various sources of bias within diffusion MRI data and processing. This is primarily intended to discard the spurious connections, and only focus on the remaining edges that usually have a higher reproducibility in the network.^[2]^ The other reason is that some of the graph theoretical metrics are only suitable for sparse networks, such as the clustering coefficient, rich-club coefficient, and small-world architecture (explained in Refs. 43, 97, 104, 105, respectively); this, however, does not constitute an appropriate basis for modifying a connectome. Notably, weak connections (often associated with long physical pathways) have been demonstrated to be important for supporting functional diversity and dynamics in the brain.^100^ Selecting an improper threshold to prune the connectome may result in losing potential realistic connec tions^106^ or in including false positives,^107^ thereby complicating the comparisons of subsequent analyses and leading to limited interpretability. As such, it is a challenge to determine a reasonable threshold that does not alter the biological features embedded originally in the structural connectome. In addition, graph theoretical metrics are generally presumed to be particularly susceptible to the network density^[3]^; such a dependency makes the selection of a threshold even more difficult. In Ref. 107, a multi-threshold technique is proposed to resolve the problem of instability induced by using a single threshold, thereby improving the power of detecting group differences in network metrics. However, pruning a connectome via thresholding is not a fundamental solution to addressing the underlying issues, since it is independent of any stage between diffusion MRI scan and connectome construction.

Advanced tractogram reconstruction methods enable the construction of dense and weighted structural connectomes.^43,59,104,108^ When analyzing this class of connectomes using graph theoretical approaches, a recent study has demonstrated that some of the most popular global network metrics are in fact not sensitive to network density (as opposed to what is typically expected in the field): no statistically significant variations are observed even when the weakest ~70–90% connections in the network are removed, with connectomes generated at different parcellation granularities.^108^ This is because the distribution of connection density in the connectome presents a heavy-tailed distribution with a large dynamic range: it expands up to five orders of magnitude differences between the weak and strong connections, with the majority of connections being weak (~80%). As such, the outcomes of weighted graph theory metrics are driven principally by those with strong connections in this type of connectome. Furthermore, it has also been demonstrated in some other species that connectomes have high connection densities.^96,105,109–112^ Together these bring about two important perspectives in connectome analysis: 1) Applying a threshold is not necessary for dense weighted connectomes, and the dense weighted version can be used to compute weighted global network metrics; 2) The current weighted graph theoretical metrics are insensitive to the contribution of weak connections to the network, while longer streamlines (e.g., interhemispheric connections) are often weaker in their strengths; this therefore implies that there remain potential limitations of the present weighted graph theoretical approaches on calculating some of the global measures.

### Graph Theoretical Analysis: From Binary to Weighted Metrics

Despite promising applications of graph theoretical analysis in connectomics, there are some caveats that need to be considered when applying such analysis to tractogram-based connectome data: Graph theoretical analysis is originally intended to investigate binary networks. As mentioned previously, “binary” means that the information of interest is only the existence or absence of a connection between nodes. The formulas for calculating network metrics are therefore primarily designed for binary systems. In general, those formulas cannot be transformed into continuous space simply by replacing the binary units of the equations by real numbers. There are certain requirements to allow this transformation to be valid: when weights are assigned to the links, those weights are physical and statistical quantities. The weighted formulas are generalizable from the binary ones only if the operations within the equations are compatible with the physical or statistical meaning of the weights.

One example is the calculation of the “distance” or so-called “shortest path.” In a binary network, determining the distance between two nodes is to find the fewest “steps” needed to travel from one node to the other. The weighted version of this is the classic “traveling salesman problem”^113^; in its simplest form, for example, the algorithm defined to seek the shortest Euclidean distance (e.g., in kilometers) between nodes (e.g., cities) is compatible with the weights of each connection, i.e., those weights are also a Euclidean distance. However, this approach becomes problematic when we apply such an algorithm to tractography-based connectome data, in which the weights of the connections are often not a distance metric but something else, such as the number of streamlines or mean fractional anisotropy. Computationally speaking, the algorithm to calculate weighted distance can always output values as long as adjacency/connectivity matrices are supplied, but those values have no physical or statistical meaning if the matrices are not compatible with the algorithms. Indeed, in practice, obtaining meaningful “distances” often requires some form of matrix conversion from a weight to a distance matrix.^19^ This is an essential step upon which a subset of network metrics is dependent, such as local and global efficiencies. It should be noted that even though this reciprocal transformation is also in itself ambiguous, the fact that this weight-to-distance conversion occurs internally within the weighted graph metric calculation function(s) could mean that researchers might be either unaware that such a step even occurs, or assume that it is an unambiguous solution.

As described above, if calculations originally intended for binary data are augmented to operate on continuous or weighted data, there are two conditions that, if satisfied, could potentially make feeding continuous or weighted data to such an algorithm feasible: 1) the weights themselves have a physical meaning; 2) the extension of the algorithm from binary to nonbinary data is consistent with a physical network interpretation; in this case, the binary version is a specific instance of the weighted version both in interpretation and outcome. The first condition can be achieved through the application of quantitative tractogram processing techniques, in which each streamline is encoded with, e.g., some cross-section area of WM fibers.^45,55^ However, if a connectome is generated based on, e.g., the mean fractional anisotropy, the fact that such data are intrinsically nonbinary does not lend any physical or statistical reasoning. Therefore, weighted global network metrics calculated from such data would not have any particular meaning.

Finally, given how prevalent graph theoretical analysis is in current connectomics research, it is necessary to be aware of any latent risks or limitations when applying the relevant techniques, particularly on tractography-based connectomes. Studies have demonstrated the potential limitations of some existing weighted network metrics calculation functions, including weighted clustering coefficient,^43^ small-world index,^43^ weighted rich-club coefficient,^104^ and weighted shortest path length.^108^

### Every Bias Correction Matters

When comparing graph theoretical metrics between groups (e.g., case vs. control), it is fundamentally important to use exactly the same underlying processing steps. However, it should not be assumed that how an individual’s connectome is constructed is unimportant as long as the same reconstruction/processing biases are present in all subjects; the presence of bias within an individual’s connectome data can still have significant impact. To this end, considerable efforts have been made to identify and reduce artifacts/biases in individual connectivity reconstruction, from the moment of MRI data acquisition through to the connectome generation. The results of group comparisons and detection of differences following the use of advanced methods that reduce biases are both more reliable and have greater interpretability than those resulting from raw data that has been processed inadequately.

## Summary

Streamline-based tractography from noninvasive diffusion MRI data is the central technique for the study of structural connectivity of the living human brain. Despite their core role and widespread applications, modern tractography techniques are still imperfect and suffer from various sources of bias.^32,33^ Any analyses derived from uncorrected, whole-brain tractography approaches will therefore also be subject to biases. Furthermore, the construction of a structural connectome involves a series of steps, and each step entails choices that can have flowthrough effects on the connectivity results. This article reviews some of the issues that need to be taken into account when applying this technique in connectomics research. It also outlines modern tractography analysis methods that have provided solutions to some of the most important sources of bias, and describes how their careful use can result in connectivity measures that better represent biological reality, which provide greatly improved input data for further connectomic analyses such as using graph theoretical approaches.

Graph theoretical analysis is one of the major techniques to quantify the topological properties of the brain network, and has prompted the rapid expansion of brain connectivity investigations. Network analysis using global metrics as derived from weighted graph theoretical methods may not always be fully appropriate. The potential challenges and limitations of the commonly-used graph theoretical methods in analyzing tractography-based structural connectomes should be considered. Although it is inherently challenging to extract the features of structural connectivity accurately with existing approaches, recognition of those challenges and important limitations of each step is critical when interpreting connectivity results, as well as to motivate new developments or improvements in connectivity mapping paradigms.

## Figures and Tables

**Figure 1:**
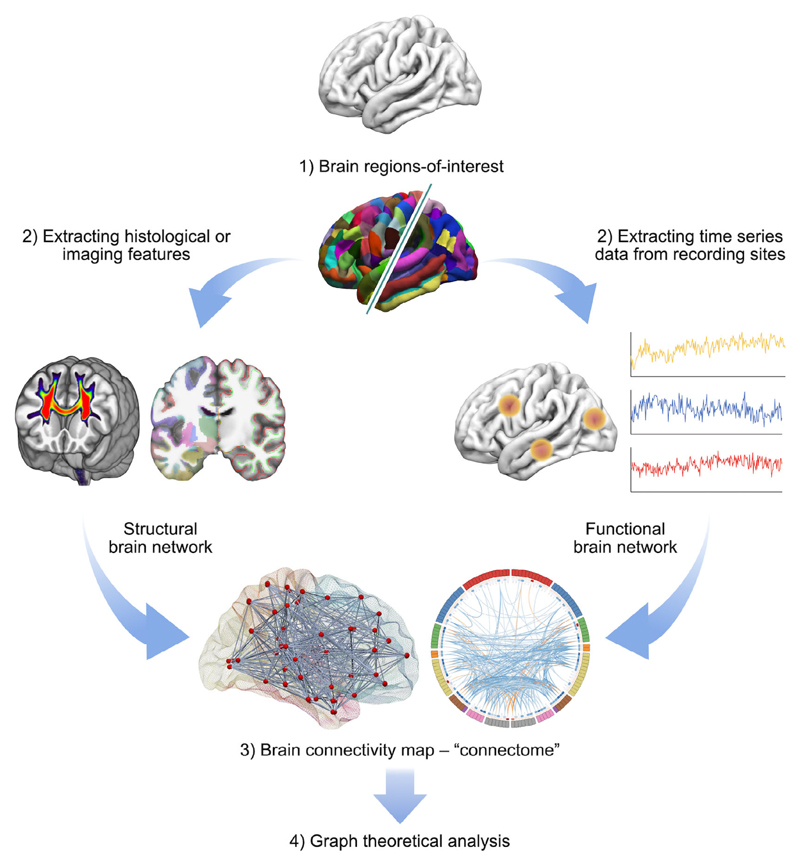
A general overview of applying graph theoretical analysis to study structural (left branch) and functional (right branch) brain networks. A network or a graph is a collection of vertices (nodes) and their pairwise links (edges). A comprehensive set of all pairwise connections in the brain defines the topology of a brain network, providing a complete connectivity diagram of all associations among nodes and edges, i.e., a connectome. There are four essential components involved in this technique: 1) Defining nodes: Nodes are brain regions-of-interest; they are typically derived from an anatomical parcellation image data but can also be from more localized areas such as using electrodes, depending on the measurement technique. 2) Defining edges: Edges are the actual measure of relations between every node pair. They can be streamline connections from diffusion MRI tractography, inter-areal brain signal correlation/synchronization from resting-state functional connectivity, or other measures such as cortical thickness. 3) Constructing a network: This step integrates all the information from nodes and edges to generate a complete map of connectivity. The simplest representation of a network is using a 2D matrix (i.e., so-called connectivity matrix), but can also be visualized in various ways. 4) Graph theoretical analysis: In the present connectomics field, the most commonly-used method to calculate the characteristics of a network is by applying graph theory, which provides various global measures about the network topology.

## Footnotes

1 It is worth emphasizing that diffusion MRI tractography does not reconstruct actual WM fibers but only streamlines (or curves in 3D space) that delineate possible WM pathways. A streamline obtained using fiber-tracking is a virtual entity, which should not be interpreted as a real nerve fiber that encompasses realistic biological and physical properties.

2 It should be noted that there is, however, no direct correspondence between accuracy and reproducibility of the reconstructed streamline connections. A white matter pathway with massive streamline connections of a high reproducibility could also be a false-positive tract.^33^

3 The fact that graph theory metrics are susceptible to network density does suggest that diminishing reconstruction bias for individual connectome construction is crucial; otherwise those metrics may only reflect how network density varies due to reconstruction bias rather than actual connectivity.
